# An environmental risk assessment of IPD079Ea: a protein derived from *Ophioglossum pendulum* with activity against *Diabrotica* spp.In maize

**DOI:** 10.1080/21645698.2023.2299503

**Published:** 2024-01-18

**Authors:** Bridget F. O’Neill, Chad Boeckman, Kristine LeRoy, Chris Linderblood, Taylor Olson, Rachel Woods, Mary Challender

**Affiliations:** a Corteva Agriscience™, Regulatory and Stewardship institution, Indianapolis, IN, USA; b Corteva Agriscience™, Regulatory and Stewardship, Johnston, IA, USA

**Keywords:** DP-915635-4 maize, genetically modified, IPD079Ea protein, non-target organism, safety assessment, zea mays L

## Abstract

Farmers in North America face significant pressure from insects in their maize fields, particularly from corn rootworm (*Diabrotica* spp.). Research into proteins capable of insecticidal activity has found several produced by ferns. One protein, IPD079Ea, was derived from *Ophioglossum pendulum* and has shown activity against corn rootworm. An environmental risk assessment was conducted for maize event DP-915635-4, which provides control of corn rootworms via expression of the IPD079Ea protein. This assessment focused on IPD079Ea and characterized potential exposure and hazard to non-target organisms (NTOs). For exposure, estimated environmental concentrations (EECs) were calculated. For hazard, laboratory dietary toxicity studies were conducted with IPD079Ea and surrogate non-target organisms. Environmental risk was characterized by comparing hazard and exposure to calculate the margin of exposure (MOE). Based on the MOE values for DP-915635-4 maize, the IPD079Ea protein is not expected to result in unreasonable adverse effects on beneficial NTO populations at environmentally relevant concentrations.

## Introduction

The United States is the world’s largest producer of maize, with 15.3 billion bushels projected to be grown in 2023.^1^ To produce this crop, farmers must contend with weather events, weed pressure, diseases, and insect pests. One of the most important groups of maize pests in the United States is corn rootworm (*Diabrotica* spp.). There are three species of economic concern, the southern corn rootworm (*Diabrotica undecimpunctata howardi*), the northern corn rootworm (*Diabrotica barberi*), and the western corn rootworm (*Diabrotica virgifera virgifera*). While the three species have slightly different ranges, and distinct adults, the larvae are indistinguishable. All three species feed on maize plants; maize foliage as adults and maize roots as larvae. The damage to the roots can restrict the growing plants’ ability to absorb water and nutrients from the soil, reduce grain production, and cause the plants to lodge, or fall over.^2^ Farmers have several tools available to them to help combat rootworm feeding, including crop rotation, insecticidal sprays and seed treatments, and genetically modified seeds. Genetically modified plants can express insecticidal proteins, historically from *Bacillus thuringiensis* (*Bt*). Wide adoption of this technology has led to reductions in the use of chemical insecticides, but also to resistance in target pests, such as corn rootworms.^3^ There is great interest in developing additional insecticidal protein options active against corn rootworms for farmers from non-*Bt* sources.

Plant extracts are increasingly being used to elucidate potential new actives useful in various agricultural applications.^4^ A family of novel proteins was identified in *Ophioglossum pendulum*, which were named IPD079. These proteins were effective against western and northern corn rootworms (Barry *et al*., in preparation). Maize (*Zea mays* L.) event DP-915635-4 (referred to as DP-915635-4 maize) expresses the IPD079Ea protein for control of corn rootworm pests. DP-915635-4 maize also expresses the PAT protein for tolerance to glufosinate herbicide, and the phosphomannose isomerase (PMI) protein from *Escherichia coli*, which was used as a selectable marker.

Risk assessments are conducted as part of the registration process for genetically- modified seed traits.^5^ Risk is defined as the likelihood and magnitude of an adverse effect (hazard) because of exposure (*e.g*., from the growth of a seed). Environmental risk is characterized by comparing hazard and exposure to calculate the MOE for beneficial NTO functional groups (a set of species within a community that have a similar ecosystem function) that may be present in the agricultural ecosystem and potentially interact with the plant by feeding upon it, or upon prey items that have fed upon it. For an environmental risk assessment for an insecticidal trait, functional groups of concern include pollinators and pollen feeders, soil-dwelling organisms, aquatic organisms, predators and parasitoids, insectivorous birds, and granivorous mammals.

Risk assessments involve a tiered testing strategy to assess hazard. Lower tier (Tier I) studies are conducted in a laboratory with individual organisms and look for adverse effects on the individual. Higher tier studies (Tier II, Tier III, or Tier IV) are conducted in the laboratory, greenhouse, or the field depending on the study, and look for field-realistic effects at the population level. With each tier there is an increase in realism and complexity of design, yet also an increase in the confounding variables that may interfere with data interpretation. Lower-tier studies (which are always conducted for a risk assessment) may trigger higher-tier studies, which are only conducted when the results from the lower tier study indicate that further hazard assessment is required.

An environmental risk assessment (ERA) for the cultivation of DP-915635-4 maize was conducted based on the risk assessment framework described by the U.S. Environmental Protection Agency.^5^ This framework is currently used to assess genetically modified (GM) (*i.e*., transgenic) crops expressing plant-incorporated protectant (PIP) traits derived from both *Bt*, and non-*Bt* sources. The framework provides a robust and suitable, science-based tool for assessing potential risks associated with genetically modified plants.^6^ Problem formulation was used to identify plausible pathways to environmental harm arising from cultivation of DP-915635-4 maize. Plausible pathways to harm exist for beneficial NTOs where exposure to the IPD079Ea protein may occur via feeding upon DP-915635-4 maize tissue or on prey and/or hosts that consume DP-915635-4 maize tissues. This ERA is focused on the insecticidal IPD079Ea protein expressed by DP-915635-4 maize and assessed the potential exposure and hazard to NTOs.

## Materials and Methods

### Non-Target Organism Exposure Assessment Overview

Worst-case EECs of the IPD079Ea protein in DP-915635-4 maize were determined using highly conservative assumptions based on the maximum concentrations of the IPD079Ea protein in relevant DP-915635-4 maize tissues to determine potential exposure for NTOs, including pollinators and pollen feeders, soil dwelling and aquatic decomposers, predators and parasitoids, granivorous mammals and insectivorous birds. These worst-case EECs were used to calculate estimated MOEs using hazard data from Tier I laboratory bioassays described below (hazard value divided by exposure value equals the margin of exposure). In cases where calculated MOEs based on worst-case EECs were less than 10, refined EECs were calculated to reflect more realistic environmental conditions and ecological processes that reduce actual NTO exposure in the field.

### Hazard Assessment

All hazard studies were conducted in compliance with Good Laboratory Practice regulations as provided in EPA 40 CFR part 160.^7^ For IPD079Ea protein bioassays, the IPD079Ea protein was expressed in *E. coli*, lyophilized, and characterized as described previously.^8,9^ The equivalence between the plant expressed and *E. coli* produced IPD079Ea protein was established.^10^ Tier I laboratory studies were conducted using representative surrogate species from several functional groups at concentrations that exceeded the EEC. For each surrogate species, the no observed effect concentration (NOEC) or noobservedeffectdose (NOED) was determined for relevant endpoints (*e.g*., mortality, adult weight) for the IPD079Ea protein.

Surrogate species were selected based on the exposure assessment, understanding of the specificity of the IPD079Ea protein^8^ as well as practical considerations (*e.g*., availability of laboratory-reared insects and established, reproducible, and robust methods).^11,12^ Surrogate species assessed included *Apis mellifera* (honey bee larvae and adults), *Folsomia candida* (springtail), *Daphnia magna*, *Chrysoperla rufilabris* (green lacewing), *Coleomegilla maculata* (pink spotted lady beetle), *Hippodamia convergens* (convergent ladybird beetle), *Dalotia coriaria* (rove beetle), *Pediobius foveolatus* (parasitic Hymenoptera), *Colinus virginianus* (Northern bobwhite quail), and *Mus musculus* (mouse); which represent functional groups of pollinators and pollen feeders, soil-dwelling detritivores, aquatic organisms, predators and parasitoids, insectivorous birds, and granivorous mammals, respectively. Insect bioassays were conducted in small environmental chambers, with temperatures ranging from 20°C to 35°C depending on the insect of interest. The light regime was maintained at either continuously dark or with a 16 L:8D photoperiod, and relative humidity was maintained above 65%, except for honey bees which varied 59–97% depending on life stage. Most of the bioassays were initiated with neonates, less than 24-hours old unless otherwise noted.

#### Honey Bee Larvae

Honey bee larvae were exposed to diets containing IPD079Ea protein (100, 250, or 500 ng IPD079Ea protein/larva/day). The bioassay was 22-days in duration and followed OECD guidance.^13^ The study included thirty-six replicates per treatment, and freshly prepared diet containing one of the following treatments (IPD079Ea protein, untreated control, or positive control, which consisted of dimethoate, a known honey bee toxicant) was provided on four consecutive days prior to pupation. The endpoints assessed included larval and pupal survival, adult emergence, and adult weight at emergence (Table 1).

**Table 1.** Laboratory studies characterizing effects of the IPD079Ea protein on representative non-target organisms.

Species (Common Name)	Class: Order: Family	Type of Study	Concentration	Endpoints Assessed	Results
**Pollinators and Pollen Feeders**					
*Apis mellifera* (Honey bee larvae)	Insecta: Hymenoptera: Apidae	22-day: IPD079Ea protein incorporated in diet	Targeted concentration of 100, 250 and 500 ng IPD079Ea protein/larva/day	Larval survival, pupal survival, adult emergence, adult weight at emergence	No adverse effects on larval survival, pupal survival, adult emergence, or adult weight at emergence were observed. The NOED is 500 ng IPD079Ea protein/larva/day.
*Apis mellifera* (Honey bee adult)	Insecta: Hymenoptera: Apidae	10-day: IPD079Ea protein incorporated in diet	Mean daily dose of 95, 230, and 430 ng IPD079Ea protein/bee/day	Adult body weight, survival	No adverse effects on adult body weight or survival were observed. The NOEC is 430 ng IPD079Ea protein/bee/day.
**Aquatic Organisms**					
*Daphnia magna*	Branchiopoda: Diplostraca: Daphniidae	21-day: IPD079Ea protein incorporated into water Static renewal	Targeted concentration of 0.1 mg IPD079Ea protein/Liter	Survival, reproduction	No adverse effects on *D. magna* survival or reproduction were observed. The NOEC is 0.1 mg IPD079Ea/L.
**Soil-Dwelling Decomposers and Detritivores**					
*Folsomia candida* (Springtail)	Collembola: Collembola: Isotomidae	28-day: IPD079Ea protein incorporated into diet	Targeted concentration of 100, 200, 400, and 800 µg IPD079Ea protein/g diet	Reproduction, survival	No adverse effect on springtail reproduction and no adverse effects on survival were observed. The NOEC is 800 µg IPD079Ea protein/g diet
**Predators and Parasitoids**					
*Chrysoperla rufilabris* (Green lacewing)	Insecta: Neuroptera: Chrysopidae	21-day: IPD079Ea protein incorporated in diet	Targeted concentration of 100, 250, and 500 µg IPD079Ea protein/g diet	Survival, pupation	Exposure to 100 µg IPD079Ea protein/g diet had no adverse effects on survival or pupation of green lacewing. The NOEC is 100 µg IPD079Ea protein/g diet
*Coleomegilla maculata* (Pink spotted lady beetle)	Insecta: Coleoptera: Coccinellidae	28-day: IPD079Ea protein incorporated in diet	Targeted concentration of 100, 200, 400, and 800 µg IPD079Ea protein/g diet	Survival, weight, days to adult emergence	No biologically relevant adverse effects on survival, weight, or number of days to adult emergence were observed. The NOEC is 800 µg IPD079Ea protein/g diet
*Dalotia coriaria* (rove beetle)	Insecta: Coleoptera: Staphylinidae	7-day: IPD079Ea protein incorporated into diet	Targeted concentration of 100, 200, 400, and 800 µg IPD079Ea protein/g diet	Survival	No adverse effect on survival was observed. The NOEC is 800 µg IPD079Ea protein/g diet
*Hippodamia convergens* (Convergent ladybird beetle)	Insecta: Coleoptera: Coccinellidae	28-day: IPD079Ea protein incorporated in diet	Targeted concentration of 100, 200, 400, and 800 µg IPD079Ea protein/g diet	Survival, weight, days to adult emergence	No adverse effects on survival, weight, or number of days to adult emergence were observed. The survival NOEC is 800 µg IPD079Ea protein/g diet
*Pediobius foveolatus* (parasitic Hymenoptera)	Insecta: Hymenoptera: Eulophidae	7-day: IPD079Ea protein incorporated into diet	Targeted concentration of 100, 200, 400, and 800 µg IPD079Ea protein/ml diet	Survival	No adverse effect on survival was observed. The NOEC is 800 µg IPD079Ea protein/ml diet
**Insectivorous Birds**					
*Colinus virginianus* (Northern bobwhite quail)	Aves: Galliformes: Odontophoridae	14-day: limit dose of IPD079Ea protein	Limit dose of 2,000 mg IPD079Ea protein/kg body weight	Survival, body weight, food consumption, behavior	No mortality, abnormal behavior or signs of toxicity were observed. The NOEC is 2,000 mg IPD079Ea protein/kg body weight.
**Granivorous Mammals**					
*Mus musculus* (Mouse)	Mammalia: Rodentia: Muridae	14-day: Acute oral assay with IPD079Ea protein	Limit dose of 5,000 mg IPD079Ea protein/kg body weight	Survival, evidence of acute oral toxicity (based on evaluation of body weight, clinical signs, and gross pathology).	No mortality or other evidence of acute oral toxicity was observed, based on evaluation of body weight, clinical signs, and gross pathology. The NOEC is 5,000 mg IPD079Ea protein/kg body weight.

#### Honey Bee Adults

Honey bee adults (≤2-d old emerged) were exposed to diets containing a mean daily dose of 95, 230, or 430 ng IPD079Ea protein/bee/day for 10 days. Bioassays followed OECD guidance.^14^ In each study, there were thirty replicates per treatment, and freshly prepared diet containing one of the following treatments (IPD079Ea protein, untreated control, or the positive control dimethoate) was provided daily. The endpoints assessed included survival and adult weight (Table 1).

Springtail adults were exposed to diets containing 100, 200, 400, or 800 µg IPD079Ea protein/g dw diet for 28 days. Springtail were housed in small wide-mouth glass jars (8 replicate jars per treatment containing a target of ten individuals each) and were provided diet containing one of the following treatments (IPD079Ea protein, untreated control, or positive control, which consisted of teflubenzuron) daily. Diets were prepared in bulk and stored frozen until use (frozen storage stability of protein was confirmed). The endpoints assessed included survival and reproduction (Table 1).

*D*. *magna* (<24-hour juveniles at initial exposure) were exposed to a IPD079Ea protein level of 0.1 mg/L for 21-days in a static-renewal test. *D. magna* were housed in glass containers and were provided food daily as per OECD guidance.^15^ The endpoints assessed included survival and reproduction (Table 1).

Green lacewing neonates were exposed to diets containing 100, 250, or 500 µg IPD079Ea per g diet for 21 days. Green lacewings were housed in 30 mL plastic cups (40 replicates per treatment) and were provided diet containing one of the following treatments (IPD079Ea protein, untreated control, or positive control, which consisted of cryolite) every other day. Diets were prepared in bulk and stored frozen until use (frozen storage stability of protein was confirmed). The endpoints assessed included survival and pupation (Table 1).

Pink spotted lady beetle neonates were exposed to diets containing 100, 200, 400, or 800 µg IPD079Ea protein/g dw diet. The study duration was 28 days or until adult emergence. *C. maculata* larvae were housed individually in Petri dishes (30 replicates per treatment) and were provided diet containing one of the following treatments (IPD079Ea protein, untreated control, or positive control, which consisted of cryolite) every 3–4 days. Diets were prepared in bulk and stored frozen until use (frozen storage stability of protein was confirmed before use). The endpoints assessed included survival, adult weight at emergence, and number of days to adult emergence (Table 1).

Convergent lady beetle neonates were exposed to diets containing 100, 200, 400, or 800 µg IPD079Ea protein/g dw diet. The study duration was 28 days or until adult emergence. *H. convergens* were housed individually in Petri dishes (30 replicates per treatment) and were provided diet containing one of the following treatments (IPD079Ea protein, untreated control, or positive control, which consisted of boric acid) every 3–4 days. Diets were prepared in bulk and stored frozen until use (frozen storage stability of protein was confirmed before use). The endpoints assessed included survival, adult weight at emergence, and number of days to adult emergence (Table 1).

Rove beetle adults were exposed to diets containing 100, 200, 400, or 800 µg IPD079Ea protein/g dw diet. The study duration was 7 days. *D. coriaria* were housed in 30 mL plastic cups (1 individual per cup), for a total of 30 individuals per treatment and were provided diet containing one of the following treatments (IPD079Ea protein treatments, untreated control, or positive control, which consisted of boric acid) daily. Diets were prepared in bulk and stored frozen until use (frozen storage stability of protein was confirmed before use). The endpoint assessed was survival (Table 1).

Parasitoid wasp adults were exposed to 30% sucrose diets containing 100, 200, 400, or 800 μg IPD079Ea protein/ml diet. The study duration was 7 days. *P. foveolatus* were housed in 30 mL plastic cups (30 replicates per treatment) and were provided diet containing one of the following treatments (IPD079Ea protein, untreated control, or positive control, which consisted of boric acid) every other day. Diets were prepared in bulk and stored frozen until use (frozen storage stability of protein was confirmed before use). The endpoint assessed was survival (Table 1).

Northern bobwhite quail were administered a nominal limit dose 2000 mg IPD079Ea protein per kg body weight for 14 days, following OCSPP Guideline 850.2100.^16^ A total of twenty birds were used in each study with five males and five females per treatment (IPD079Ea protein and the negative control bovine serum albumin). The endpoints assessed included mortality, body weight, food consumption, and abnormal behavior (Table 1).

Mice were orally exposed at a limit dose of 5000 mg IPD079Ea protein/kg body weight for 15 days, following OECD, Section 4 (Part 423),^17^ with the following exceptions: number of animals (*n* = 6 males and *n* = 6 females per treatment), addition of a vehicle control (deionized water) group, and addition of a Bovine Serum Albumin (BSA) protein control group. Male and female (nulliparous and non-pregnant) Crl:CD1(ICR) mice were used in the study. The endpoints assessed included mortality, evidence of oral toxicity based on body weight, clinical signs, and gross pathology (Table 1).

### Statistical Analysis

Statistical analyses of surrogate insect species other than honey bee, *D. magna*, and quail were conducted using SAS™ software, Version 9.4 (SAS Institute Inc., Cary, NC, USA) separately for each study and each measured endpoint. Statistical analysis of survival data was conducted using Fisher’s exact test (SAS PROC MULTTEST) to determine if the survival observed for the IPD079Ea protein treatments were less than the survival observed with the untreated control treatment included in each study. The statistical analysis methods used to assess the weight of surviving insects were dependent upon the validity of statistical assumptions for each data set. For some experiments, the normality assumption was satisfied by the data distributions of the test and control entries, thus an analysis of variance (SAS PROC GLIMMIX) was conducted to assess if the test diet caused growth inhibition. If the normality assumption was not satisfied, SAS PROC NPAR1WAY was used to conduct both Wilcoxon two-sample and Siegel – Tukey test. The Siegel – Tukey test was conducted to further assess for differences in scale between the two treatments^18^ and to determine if exposure to the test diet caused a developmental delay. For days to adult emergence, in all cases the normality assumption was not satisfied; thus, both Wilcoxon two-sample and Siegel – Tukey tests were conducted. For reproduction, a generalized linear mixed model (SAS PROC GLIMMIX) was fit to the reproduction data assuming a Poisson distribution and a fixed effect of treatment. For each of these tests, all *P*-values were considered significant if < 0.05.

Statistical analyses of honey bee and *D. magna* data were conducted using either CETIS Version 1.8 or 1.9^19^ for each study and each measured endpoint. All comparisons for determination of a NOED and LOED were made at ≥ 95% level of certainty (*p* < .05) and compared on a per replicate (individual well or bee) basis. Statistical analysis of survival or emergence data was conducted using Fisher’s Exact Test with Bonferroni-Holm’s Adjustment. Weight data were first evaluated by conducting Shapiro-Wilk’s Test to assess normality of the distribution and Bartlett’s Test to assess homoscedasticity, and then the results of these tests were used to select the statistical method used in comparisons and determination of NOED and LOED values. For IPD079Ea honey bee larval weight data, Dunn’s Test with Bonferroni- Holm’s Adjustment was used for comparisons. For IPD079Ea adult weights measured in the larval study, Dunnett’s multiple comparison test was used for comparisons.

Statistical analyses of Northern bobwhite quail data were conducted using SAS v9.4. After confirming that statistical assumptions were satisfied, appropriate t-tests were run to determine whether there were differences in weight between sexes and between treatments prior to treatment. Mean measured body weights, calculated body weight change and weekly feed consumption per bird per day were similarly analyzed at the end of the study. Nonparametric data was analyzed using the Wilcoxon’s Rank Sum Test [α = 0.05;.^20,21^]. The nominal oral limit dose assessed in this limit test and corresponding mortality data were used to empirically estimate whether the median lethal dose (LD_50_) and the No Observed Effect Concentration (NOEC) were greater or less than the highest nominal concentration assessed. A similar approach was used to establish the LD_50_ for the mouse study.

### Expression of IPD079Ea Protein

A study was conducted during the growing season at five sites in commercial maize-growing regions of the United States and one site in Canada, for six sites total. Tissue samples were collected from various maize tissues (leaf, root, pollen, forage, and grain) at multiple growth stages. Tissue samples were analyzed for IPD079Ea protein concentrations using quantitative enzyme-linked immunosorbent assay (ELISA) methods.^22^ IPD079Ea protein concentrations from this study were used to evaluate beneficial NTO exposure to the IPD079Ea protein resulting from the cultivation of DP-915635-4 maize.

### Estimated Environmental Concentration Calculations

Adult honey bees feed on pollen from a variety of different plant species as a major source of protein and consume nectar for sugar and carbohydrates.^23,24^ For these calculations, it is assumed that honey bees are exposed to 100% of the maximum concentration of the IPD079Ea protein in DP-915635-4 maize pollen. The amount of pollen consumed by one honey bee larva has previously been estimated to be approximately 1.5–2.0 mg over the course of development,^25^ and the amount of pollen consumed by one honey bee adult has previously been estimated to be 3.4–4.3 mg per day.^26^ Because pollen is a primary food source, honey bees are likely to be present and actively foraging during corn pollen shed.^26^ However, population level exposure to DP-915635-4 maize pollen is expected to be low as maize pollen is not a preferred food source and not expected to be the only dietary component consumed.

Soil-dwelling decomposers and detritivores are most likely to consume senescent maize tissues that are incorporated into the soil post-harvest.^27^ The worst-case EEC for soil-dwelling organisms was calculated based on the maximum concentration of the IPD079Ea protein in V9 root tissue. While soil-dwelling organisms ingest senescent tissues, V9 dry weight root tissue values are used as a worst-case exposure for conservative calculations as the value in V9 tissue is the starting point with concentrations reducing as senescing tissues degrade.

The worst-case EEC for aquatic organisms to the IPD079Ea protein in DP-915635-4 maize tissues was estimated using the EPA standard agricultural field-farm pond model (also called the US EPA standard pond model^28^). The EPA standard agricultural field-farm pond model provides estimates for predicting pesticide runoff concentrations and uses the assumptions that runoff from a 10-hectare (ha) field is deposited in a 1-ha pond (2 meters deep; equivalent to 20,000,000 L of water). This approach has previously been adapted to model a conservative exposure scenario for aquatic NTOs to newly expressed proteins in GM crops.^29–31^

The worst-case EECs for aquatic organisms can be calculated based on the greatest above ground tissue concentration (greatest value from any growth stage) of IPD079Ea protein dry weight. A refined EEC for aquatic organisms was also considered by using the greatest mean value for any above ground tissue type.

The worst-case EEC for predators and parasitoids and for insectivorous birds assumes: 1) that 100% of the protein in the GM plant transfers to the prey or host and then subsequently is transferred to the predator or parasitoid (no degradation or loss); and 2) that predators and parasitoids are exposed to the maximum concentration of the IPD079Ea protein expressed in tissue (maximum from any above-ground dry weight plant tissue and from any growth stage). Several factors will reduce the actual exposure of predators and parasitoids to IPD079Ea protein below the worst-case EEC (for example, duration of pollen shed, other pollen sources, other diet components, etc.).

For wild mammals that consume grain, a worst-case EEC assumes consumption of a diet that contains 73% maize grain,^32^ and is based on the maximum concentration of the IPD079Ea protein in maize grain. A daily dietary dose (DDD) for wild rodents can be calculated,^30^ which accounts for food intake, body weight, and the IPD079Ea protein concentration in dry weight grain.

### Margin of Exposure Calculations

For each surrogate species, the NOEC or NOED was determined for relevant endpoints (*e.g*., mortality, adult weight) for the IPD079Ea protein and compared to the worst-case EEC or refined EEC to determine the MOE. For this comparison, the relevant endpoint value was divided by the chosen EEC. An MOE that exceeds 10X is considered conservative and is indicative of minimal risk under realistic environmental conditions.^33^ All MOE calculations were conducted based on the weight concentration of the IPD079Ea protein in DP-915635-4 maize tissues. Wet weights were used for calculations for honey bee adult and larvae, green lacewing, and the parasitic wasp. Dry weights were used for the other organisms, as the diets in the exposure studies were lyophilized and dry weight recorded. The dry weight concentrations are considered high estimates, since in reality NTOs would be exposed to levels comparable to fresh weight levels.^34^

#### Honey Bees

For the MOE calculation, it is assumed that honey bee adult and larvae are exposed to 100% of the maximum concentration of the IPD079Ea protein in DP-915635-4 maize pollen.

#### Non-Target Lepidoptera

Most non-target Lepidoptera larvae do not feed on pollen directly, but indirectly may be exposed to pollen as they feed on host plants. Therefore, the degree of potential exposure of non-target Lepidoptera to DP-915635-4 maize pollen will depend on the presence of host plants in and adjacent to DP-915635-4 maize fields, as well as the timing and rate of maize pollen deposition. Four lepidopteran species from four different families (European corn borer (*O. nubilalis;* Crambidae), codling moth (*C. pomonella*; Tortricidae), painted lady (*V. cardui*; Nymphalidae), and corn earworm (*H. zea*; Noctuidae)), were exposed to diet containing a target concentration of 800 µg IPD079Ea protein/g diet and assessed as part of the spectrum of activity assessment of the IPD079Ea protein (Boeckman, 2020). For the MOE calculation, it is assumed that non-target Lepidoptera are exposed to 100% of the maximum concentration of the IPD079Ea protein in DP-915635-4 maize pollen.

#### Soil-Dwelling Organisms

The IPD079Ea protein in DP-915635-4 maize may enter the soil through root exudates, root sloughing, pollen deposition, and post-harvest plant tissue decomposition. Soil-dwelling organisms may be exposed to the IPD079Ea protein via ingestion of DP-915635-4 senescent maize tissues (detritivores).

One factor to consider in the exposure assessment of soil-dwelling organisms is the stability of the IPD079Ea protein in soil. A laboratory study was conducted to characterize the fate of the IPD079Ea protein in different soil types. Dissipation of the IPD079Ea protein in loam, sandy loam, and silt loam was determined using a sensitive insect bioassay and western blot analyses.^35^ Dissipation of IPD079Ea protein in these diverse soil types occurred within 7 days, and the protein is therefore unlikely to persist or accumulate in the field where DP-915635-4 maize is cultivated.

For these calculations, the worst-case EEC for soil-dwelling organisms can be calculated based on the maximum concentration of the IPD079Ea protein in V9 root tissue.

#### Aquatic Organisms

The worst-case EEC for aquatic organisms to the IPD079Ea protein in DP-915635-4 maize tissues was estimated using the EPA standard agricultural field-farm pond model (also called the US EPA standard pond model^28^). The EPA standard agricultural field-farm pond model provides estimates for predicting pesticide runoff concentrations and uses the assumptions that runoff from a 10-hectare (ha) field is deposited in a 1-ha pond (2 meters deep; equivalent to 20,000,000 L of water).For these calculations, it is assumed that one maize plant weighs 0.3 kg dry weight; maize density is 75,000 plants/ha; equivalent to 22,500 kg plant tissue/ha.^29^ It is also assumed that the IPD079Ea protein in DP-915635-4 maize tissue are freely soluble and instantaneously bioavailable, and that no protein degradation occurs in the field or the pond. The greatest leaf tissue concentration (greatest value from any growth stage) of the IPD079Ea protein is used to be most conservative and used to derive a worst-case EEC. The greatest mean leaf tissue concentration (greatest mean value from any growth stage) of the IPD079Ea protein is used to derive a refined EEC.

#### Predators and Parasitoids

A predator or parasitoid may be exposed to the IPD079Ea protein via consumption of prey or host that has previously consumed tissue from a DP-915635-4 maize plant. Because a predator or parasitoid does not feed directly on the maize plant, one factor to consider in the exposure assessment for predators or parasitoids is the amount of IPD079Ea protein that transfers and potentially accumulates in the prey or host. Secondary exposures via prey or host are influenced not only by the rates of ingestion, digestion and excretion of plant material by the prey or host,^33^ but also by the stability of the IPD079Ea protein within them.

Worst-case EECs were calculated for predators and parasitoids exposed to the IPD079Ea protein via prey/host. It is assumed that 100% of the protein in the GM plant transfers to the prey/host and then subsequently is transferred to the predator/parasitoid (no degradation or loss). For these calculations, predators/parasitoids are exposed to the maximum concentration of the IPD079Ea protein expressed in tissue (maximum from any above-ground plant tissue and from any growth stage) and transferred to the predator/parasitoid.

#### Green Lacewing

Green lacewing larvae are generalist predators that may inhabit maize fields and field margins. The route of exposure of green lacewing larvae to the IPD079Ea protein in DP-915635-4 maize plants is via secondary transfer via prey (predatory route). A limited number of studies indicate adult green lacewing may also feed on tiny amounts of plant tissue. However, because the larval stage is considered the most sensitive, an EEC based on larval exposure to the IPD079Ea protein via secondary transfer via prey is considered most protective of the population. The potential route of exposure through plant tissue is also covered here with the assumption that 100% of the IPD079Ea protein is transferred from the plant, through the prey, to the predator, without degradation.

#### Ladybird Beetles

A limited number of coccinellid species may feed directly on maize pollen (pollinivorous species); however, the majority of coccinellids may only incidentally consume pollen when consuming other food. For coccinellids that either directly or indirectly consume DP-915635-4 maize pollen, the worst-case EECs is considered low relative to other potential routes of exposure. Several factors will further reduce the actual exposure of pollen feeding non-target coccinellids to the IPD079Ea protein below these worst-case EECs (*e.g*., duration of pollen shed, other pollen sources, other diet components, etc.).

In addition to the pollen route of exposure, non-target coccinellids may be exposed to the IPD079Ea protein from DP-915635-4 maize via predatory or herbivory routes of exposure. Predatory and/or herbivorous coccinellids would be exposed to a higher concentration of the IPD079Ea protein, relative to pollen exposure.

Ladybird beetles are generalist predators that may inhabit maize fields and field margins. The route of exposure to the IPD079Ea protein in DP-915635-4 maize is via secondary transfer via prey (predatory route). Ladybird beetles may also feed on small quantities of plant tissue. This potential route of exposure is also addressed with the assumption that 100% of the IPD079Ea protein is transferred from the plant, through the prey, to the predator, without degradation.

#### Parasitic Hymenoptera

Parasitic Hymenoptera may be exposed to the IPD079Ea protein in DP-915635-4 maize via secondary transfer (predatory species) if their larval hosts are feeding on DP-915635-4 tissue. For the purposes of calculating an EEC, the predatory route of exposure was used to derive the EECs.

#### Rove Beetle

Rove beetle are generalist predators that may inhabit maize field and field margins. The route of exposure of rove beetle larvae and adults to the IPD079Ea protein in DP-915635-4 maize plants is via secondary transfer via prey (predatory route).

#### Insectivorous Birds

Some wild birds are insectivorous and could be exposed to the IPD079Ea protein via prey (tri-tropic transfer via the predatory route). The factors that may limit the potential exposure of wild birds to the IPD079Ea protein via prey are discussed above for predators and parasitoids. In these calculations it is assumed that predators are exposed to the maximum concentration of the IPD079Ea protein expressed in tissue (maximum from any above-ground plant tissue and from any growth stage). And it is also assumed that 100% of the protein in the GM plant transfers to the prey and then subsequently is transferred to the predator (no degradation or loss).

#### Granivorous Mammals

Granivorous wildlife (*e.g*., rodents) may be exposed to the IPD079Ea protein by feeding on DP-915635-4 maize grain. Several factors may limit the potential exposure of granivorous mammals to the IPD079Ea from DP-915635-4 maize. Wild rodents are unlikely to consume only maize grain, as they typically feed on a variety of cereal seeds. Also, the concentration of the IPD079Ea in DP-915635-4 maize grain is low (maximum concentration in grain is 0.36 µg/g.^22^

For these calculations, maximum concentration of the IPD079Ea protein in DP-915635-4 maize grain (R6) was used. It was assumed that 73% of wild mammal diet is maize grain.^32^ A daily dietary dose (DDD) was calculated, which accounts for food intake, body weight, and the IPD079Ea protein concentration in grain, where DDD = FIR/BW * C.^32,36^ The worst-case FIR/BW ratio for seed-eating rodents is 0.33 for the harvest mouse (*Micromys minutus*).^32^

## Results and Discussion

Chemical pesticide risk assessments have a robust system of guidelines for what studies need to be conducted to address hazard for various functional groups, and how exposure should be calculated for each of those groups. Hazard and exposure are then compared to assess risk. Plant-Incorporated Protectants (PIPs) follow similar guidance, with the understanding that exposure will be more limited than that found for chemical applications. Risk must still be assessed for reduced risk compounds, such as PIPs. Assessments for pesticidal products have safety margins built into them, so an acceptable risk is only concluded if the level at which a hazard might be seen in a Tier I study is well above the amount an organism could potentially be exposed to in the environment.^5^ For PIPs, the level regarded as acceptable is ten times, in that studies try to measure any potential hazard to NTOs with exposure to the protein at least 10-times the level that is the EEC.^37^ The MOE values in the ERA then have a certain amount of conservatism built into them, resulting in confidence in the safety for NTOs in the environment.

### Non-Target Organism Hazard Assessment

Hazard assessments should be conducted using a tiered testing system.^38–40^ Tier I or II studies are laboratory-based bioassays designed to provide large margins of exposure, targeting concentrations that are at least 10X the EEC.^33,40^ If no significant hazard (*i.e*., greater than 50% mortality) is observed at high laboratory exposures, then the risk of adverse effects at environmentally relevant concentrations is likely to be low.^33^ Only when the early tier hazard study detects an effect is a higher tier study warranted,^41^ taking into account the level of concern associated with the environmental hazard, potential mitigation options to alleviate the concern, and the ability to conduct more complex higher tier studies (*e.g*..^33,39^

Problem formulation and the exposure assessments were used to guide hazard testing. Laboratory studies were conducted using representative surrogate species at concentrations exceeding the worst-case EECs to assess for potential adverse impacts of the IPD079Ea protein. Selection criteria for relevant surrogate species included phylogenetic relation to the target insects, ecological function, presence in the agroecosystem, and practical considerations such as availability of the test species, amenability to testing, and availability of standard test methods.^11,29,42,43^ Organisms selected for these studies were comprised of species representative of pollinators and pollen feeders, soil-dwelling organisms, predators and parasitoids, granivorous mammals and insectivorous birds.

The NTO studies conducted for the IPD079Ea protein were summarized and the NOEC or NOED are reported below in Table 1. The MOE for the IPD079Ea protein using worst-case or refined EECs for DP-915635-4 maize tissues are also included in the summaries below and in Table 2.

**Table 2.** Worst-case and refined EEC and MOE for representative non-target organisms exposed to IPD079Ea protein from DP-915635-4 maize.

Species (Common Name)	Worst-case EEC	Refined EEC	Hazard study result	MOE for DP-915635-4 Maize
**Pollinators and Pollen Feeders**				
*Apis mellifera* (Honey bee larvae)	Larvae eat 2 mg of pollen; Maximum pollen concentration. EEC = 2.6 µg/g	No refinement to worst-case EEC considered	NOED is 500 ng IPD079Ea protein/larval.	192X based on a worst-case EEC.
*Apis mellifera* (Honey bee adult)	Assume adults consume 4.3 mg of pollen; Maximum pollen concentration. EEC = 5.59 µg/g	No refinement to worst-case EEC considered	NOEC is 430 ng IPD079Ea protein/bee/day.	77X based on a worst-case EEC.
Non-target Lepidoptera	Maximum pollen concentration. EEC = 1.3 µg/g	No refinement to worst-case EEC considered	NOEC is 800 µg/IPD079Ea protein/g diet.	615 X based on a worst-case EEC
**Soil-Dwelling Decomposers and Detritivores**				
*Folsomia candida* (Springtail)	Maximum concentration in V9 maize root tissue. EEC = 33 µg/g	No refinement to worst-case EEC considered	NOEC is 800 µg IPD079Ea protein/g diet	24 X based on a worst-case EEC.
**Aquatic Organisms**				
*Daphnia magna*	EPA standard pond model (greatest above ground tissue concentration across any growth stage). EEC = 0.029 mg/L	EPA standard pond model (greatest mean above ground tissue concentration across any growth stage). EEC = 0.0093 mg/L	NOEC is 0.1 mg IPD079Ea protein/L	3.4X based on a worst-case EEC 10.8X based on a refined EEC
**Predators and Parasitoids**				
*Chrysoperla rufilabris* (Green lacewing)	Maximum concentration in above-ground tissue (greatest expressing tissue across the growing season). EEC = 2.6 µg/g	No refinement to worst-case EEC considered	NOEC is 100 µg IPD079Ea protein/g diet	38.5 X based on a worst-case EEC.
*Coleomegilla maculata* (Pink spotted lady beetle)			NOEC is 800 µg IPD079Ea protein/g diet	308 X based on a worst-case EEC.
*Dalotia coriaria* (Rove beetle)			NOEC is 800 µg IPD079Ea protein/g diet	308 X based on a worst-case EEC.
*Hippodamia convergens* (Convergent ladybird beetle)			Survival NOEC is 800 µg IPD079Ea protein/g diet	308 X based on a worst-case EEC.
*Pediobius foveolatus* (parasitic Hymenoptera)			NOEC is 800 µg IPD079Ea protein/ml diet	308 X based on a worst-case EEC.
**Insectivorous Birds**				
*Colinus virginianus* (Northern bobwhite quail)	Maximum concentration in above-ground tissue (greatest expressing tissue across the growing season). EEC = 2.6 µg/g	No refinement to worst-case EEC considered	The NOEC is 2,000 mg IPD079Ea protein/kg body weight.	769 X based on a worst-case EEC.
**Granivorous Mammals**				
*Mus musculus* (Mouse)	The worst-case EEC for wild mammals is based on the daily dietary dose (DDD) via consumption of R6 grain. EEC = 0.087 mg/kg body weight.	No refinement to worst-case EEC considered	NOEC is 5,000 mg IPD079Ea protein/kg body weight.	57,471 X based on a worst-case EEC.

^1^All MOEs are calculated based on tissue dry weight (DW).

### Pollinators

Pollinators and pollen feeders can be exposed to the IPD079Ea protein in DP-915635-4 maize pollen by directly feeding on maize pollen or by incidentally feeding on maize pollen (*i.e*., pollen deposited on host plant leaves). Laboratory studies were conducted to assess hazard to the pollinator and pollen feeder functional group.

#### Honey Bee

Studies were performed to evaluate the dietary effect of the IPD079Ea protein on honey bee larvae and adults. For honey bee larvae, no effects on larval survival, pupal survival, adult emergence, or adult weight at emergence were observed. The NOED for honey bee larvae is therefore the highest dose assessed, 500 ng IPD079Ea protein/larva/day. The positive control resulted in 67% mortality.

For honey bee adults, no effects on adult body weight or survival were observed. The NOEC for honey bee adults is therefore the highest concentration assessed, 430 ng IPD079Ea protein/bee/day. The positive control resulted in 97% mortality.^44^

The concentration of the IPD079Ea protein in pollen ranged from 0.58–1.3 µg/g pollen dry weight.^22^ For non-target pollinators and pollen feeders, the worst-case EEC assumes that pollen feeders and pollinators are exposed to the maximum concentration of IPD079Ea protein in pollen. Based on the maximum concentration of IPD079Ea in DP-915635-4 maize pollen, and the maximum amount of pollen consumed by honey bee larvae (2.0 mg) and adults (4.3 mg), the worst-case EEC for honey bee is 2.6 ng/larvae and 5.59 ng/adult bee (Table 2).

The larval MOE, based on a NOED of 500 ng IPD079Ea protein/larvae, is 192X the worst-case EEC for honey bee larvae exposed to the IPD079Ea protein in DP-915635-4 maize pollen (Table 2). The adult MOE, based on a NOEC of 430 ng IPD079Ea protein/bee/day, is 77X the worst-case EEC for honey bee adults exposed to the IPD079Ea protein in DP-915635-4 maize pollen (Table 2). No hazard was detected In the hazard assessments on honey bee larvae after exposure to 192X the worst-case EEC of IPD079Ea protein. No hazard was detected on honey bee adults after exposure to 77X the worst-case EEC of IPD079Ea protein. The overall MOE values indicate that the IPD079Ea protein in DP-915635-4 maize is not expected to result in adverse effects to honey bees at environmentally realistic concentrations.

#### Non-Target Lepidoptera

Based on the concentration of the IPD079Ea protein in DP-915635-4 maize pollen, the worst-case exposure of non-target Lepidoptera to the IPD079Ea protein was estimated to be 1.3 µg IPD079Ea protein/g pollen (Table 2). For the studies previously conducted with species of Lepidoptera, no adverse effects on survival were observed at 800 µg IPD079Ea protein/g diet, the highest concentration for any of the tested species (Boeckman, 2020,^45^ which is a concentration that is 615X greater than the worst-case EECs for non-target Lepidoptera. This worst-case exposure analysis assumes that the non-target Lepidoptera is only eating pollen, does not consider pollen density settling on host plants for non-target Lepidoptera, nor does it account for location of the host plant relative to a DP-915635-4 maize field. These additional factors would further reduce the potential exposure of non-target Lepidoptera to the IPD079Ea protein in DP-915635-4 maize; however, given the negligible exposure derived from this overly simple exposure model and the empirically demonstrated lack of adverse effects on non-target Lepidoptera from IPD079Ea protein, no further exposure refinements were considered necessary.

### Soil-Dwelling Organisms

A study was performed to evaluate the dietary effect of the IPD079Ea protein on springtail, which was selected to represent a non-target detritivore. No adverse effects on springtail reproduction and survival were observed. The positive control resulted in 100% mortality.^46^ The MOE based on a NOEC of 800 µg IPD079Ea protein/g diet (the highest concentration assessed), is 24X the worst-case EEC (Table 2).

For non-target detritivores, no adverse effects were observed in the hazard assessment of a representative species, *F. candida*. The NOEC is 24X the worst-case EEC of the IPD079Ea protein; therefore, the risk to non-target detritivores from IPD079Ea protein in DP-915635-4 maize is negligible.

### Aquatic Non-Target Organisms

Potential exposure of non-target aquatic organisms to plant incorporated protectants in GM crops has been considered, with movement of plant tissue identified as the most likely route of exposure for aquatic organisms.^47^ Although aquatic habitats may be located near agricultural areas, exposure of aquatic organisms to biotech crops is limited temporally and spatially^27^ and aquatic exposure to transgenic maize is extremely small.^41^ The specificity and environmental fate of the IPD079Ea protein, as well as the worst-case assumptions about potential input of maize tissue in aquatic environments, can be used to help inform the risk assessment for aquatic organisms.^29^

Based on the assumptions of the EPA farm pond model, the worst-case EEC for aquatic organisms is 0.029 mg/L for the IPD079Ea protein when considering the maximum concentration of IPD079Ea protein observed in any above ground tissue. A refined EEC was calculated using the greatest mean IPD079Ea protein concentration for any above ground tissue. The refined EEC for aquatic organisms is 0.0093 mg/L (Table 2).

In the *D. magna* study, no adverse effects on reproduction or survival were observed. The MOE based on a NOEC of 0.1 mg IPD079Ea protein/L (the highest concentration assessed) is 3.4X the worst-case EEC and 10.8X the refined EEC (Table 2).

The worst-case EEC is conservative in that, not all of the plant biomass would be transported into an adjacent aquatic system, the IPD079Ea protein has been shown to degrade rapidly in soil^35^ and similar degradation would be expected in aquatic systems, and not all the IPD079Ea protein would be freely soluble and immediately available to aquatic organisms. The IPD079Ea protein would be expected to be degraded by abiotic factors (photodegradation, pH, and temperature) and by biotic factors (microbial degradation) thus actual exposure would be significantly less. Given the conservative assumptions used to estimate aquatic exposure and additional mitigating factors such as protein degradation, the actual exposure of aquatic organisms to IPD079Ea protein will be lower than modeled here. Therefore, the risk to non-target aquatic organisms from IPD079Ea protein in DP-915635-4 is negligible.

### Predators and Parasitoids

Predators and parasitoids are an important group of NTOs that may be found within the agroecosystem. Laboratory studies were conducted to assess the dietary effect of IPD079Ea protein to this functional group using suitable surrogate species. For incidental coccinellid pollen feeders, two routes of exposure were considered, via pollen feeding and as predators. As the predatory route of exposure was deemed to result in greater EECs relative to feeding on pollen, the risk to coccinellids will be characterized via the predatory route of exposure.

#### Green Lacewing

While there were no adverse effects on pupation and no significant effects on mortality at the 250 µg treatment level in the green lacewing study, there was increased mortality. There were significant adverse effects on mortality, pupation rate, and days to pupation at the 500 µg treatment level. No adverse effects on survival or pupation of green lacewing were observed at 100 µg IPD079Ea/g diet. The positive control resulted in 95% mortality. The MOE, based on a NOEC of 100 µg IPD079Ea protein/g diet, is 38X the worst-case EEC for predators and parasitoids exposed to the IPD079Ea protein in DP-915635-4 maize via prey (Table 2).

#### Pink spotted lady Beetle

In the hazard assessment exposure to 800 µg IPD079Ea protein/g diet had no biologically relevant adverse effects on survival, weight, or days to adult emergence. There was a significant difference on emergence for two of the treatments (200 µg/g and 800 µg/g) using the Wilcoxon test (P-values = 0.0308 and 0.0111 for Treatments 3 and 5, respectively). But the data were reanalyzed using the Siegel-Tukey test (P-values = 0.5827 and 0.4641 for 200 µg/g and 800 µg/g, respectively) which did not show considerable evidence that the scales of the populations differed. The significant difference identified with the Wilcoxon test can be interpreted as a difference between the medians (emergence at day 13 for untreated diet and day 14 for treatments 200 µg/g and 800 µg/g). The observed one-day difference in emergence between 200 µg/g and 800 µg/g and untreated diet is not considered biologically relevant in this bioassay given the lack of dose-dependent response observed in this study. The positive control resulted in 95.8% mortality.^48^ The MOE, based on a NOEC of 800 µg IPD079Ea protein/g diet, is 308X the worst-case EEC for predators and parasitoids exposed to the IPD079Ea protein/g in DP-915635-4 maize via prey (Table 2).

#### Convergent Ladybird Beetle

No adverse effects on survival, weight, or number of days to adult emergence of *H. convergens* were observed at the highest concentration assessed 800 µg IPD079Ea protein/g diet. The positive control resulted in 100% mortality. The MOE, based on a NOEC of 800 µg IPD079Ea protein/g diet, is 308X the worst-case EEC for predators and parasitoids exposed to the IPD079Ea protein in DP-915635-4 maize via prey (Table 2).

#### Parasitic Hymenoptera

In the parasitoid wasp study, no adverse effects on survival were observed. The positive control resulted in 96.7% mortality. The MOE, based on a NOEC of 800 µg IPD079Ea protein/ml diet (the highest concentration assessed), is 308X the worst-case EEC for predators and parasitoids exposed to the IPD079Ea protein in DP-915635-4 maize via prey (Table 2).

#### Rove Beetle

In the rove beetle study, no adverse effects on survival were observed. The positive control resulted in 96.7% mortality. The MOE, based on a NOEC of 800 µg IPD079Ea protein/g diet (the highest concentration assessed), is 308X the worst-case EEC for predators and parasitoids exposed to the IPD079Ea protein in DP-915635-4 maize via prey (Table 2).

Five surrogate species representing the predator and parasitoid functional group were assessed: two Coccinellidae, one Neuroptera, one Hymenoptera, and one Staphylinidae. For *C. maculata* and *H. convergens*, no biologically relevant effects on survival, weight, or number of days to adult emergence were observed after exposure to 308X the worst-case EEC. No effects on survival were observed for *P. foveolatus* or *D. coriaria*, after exposure to 308X the worst-case EEC. The overall MOE values for the representative surrogate Coccinellidae, Hymenoptera, and Staphylinidae species indicate that the IPD079Ea protein in DP-915635-4 maize is not expected to result in adverse effects to predators and parasitoids at environmentally realistic concentrations.

No effects on survival or pupation were observed for *C. rufilabris*, after exposure to 38.5X the worst-case EEC. Activity of the IPD079Ea protein was observed in *C. rufilabris* at the highest concentrations assessed in the hazard study (mortality 28.2% at 250 µg IPD079Ea/g diet and 45% at 500 µg IPD079Ea/g diet). However, these concentrations are extremely high (100 – 200X), relative to the worst-case EEC. Since this high exposure is unlikely to occur in the environment, the risk to non-target *C. rufilabris* is low. Furthermore, as described in the exposure assessment, several factors will reduce the actual exposure of predators and parasitoids species to the IPD079Ea protein via the predator route, below the worst-case EEC. These factors include: 1) food and prey choice (*e.g*., not all prey consumed will have consumed DP-915635-4 maize tissue; predator or parasitoid species may feed on pollen or prey); 2) degradation of the IPD079Ea protein in prey (the IPD079Ea protein will likely not persist in prey); 3) and fresh weight vs. dry weight concentrations (all MOE calculations were conducted based on the dry weight concentration of IPD079Ea protein in DP-915635-4 maize tissues). The dry weight concentrations are considered high estimates, since in reality NTOs would be exposed to levels comparable to fresh weight levels.^34^ Therefore, no biologically relevant adverse effects are expected on predators or parasitoids, including *C. rufilabris*, due to cultivation of DP-915635-4 maize.

### Insectivorous Birds – Bobwhite Quail

A laboratory study was conducted to assess the dietary effect of the IPD079Ea protein to insectivorous birds using a suitable surrogate species. No mortality, abnormal behavior, or signs of toxicity were observed. The MOE, based on a NOEC of 2,000 mg IPD079Ea protein/kg body weight (the highest concentration assessed), is 769X the worst-case EEC for wild birds that are exposed via an insectivorous route of exposure (predator) (Table 2).

*C*. *virginianus* was assessed against IPD079Ea protein and served as a surrogate insectivorous bird. No hazard was detected in the hazard assessment after exposure to 769X the worst-case EEC. The overall MOE value for insectivorous birds indicates that the IPD079Ea protein in DP-915635-4 maize is not expected to result in adverse effects at environmentally realistic concentrations.

### Granivorous Mammals – Mouse

A laboratory study was conducted to assess the dietary effect of the IPD079Ea protein to non-target granivorous mammals, using a suitable surrogate species. No mortality or other evidence of acute oral toxicity was observed, based on evaluation of body weight, clinical signs, and gross pathology. The NOEC for *M*. *musculus* was determined to be the highest concentration assessed, 5,000 mg IPD079Ea/kg body weight.^49^ The MOE, based on 5,000 mg IPD079Ea/kg body weight, is 57471X the worst-case EEC for wild mammals exposed via the grain feeding route of exposure (Table 2).

*M*. *musculus* was assessed against IPD079Ea protein and served as a surrogate granivorous mammal. No hazard was detected in the hazard assessment after exposure to 57471X the worst-case EEC. The overall MOE value for granivorous mammals indicates that the IPD079Ea protein in DP-915635-4 maize is not expected to result in adverse effects at environmentally realistic concentrations.

The IPD079Ea protein has been shown to have a narrow spectrum of activity. The MOE values for DP-915635-4 maize indicate that the IPD079Ea protein is not expected to result in adverse effects to NTO populations at environmentally relevant concentrations. The approach used to assess environmental risk followed the EPA’s risk assessment framework, which is robust and suitable for assessing risk of non-*Bt* proteins. In conclusion, no unreasonable adverse effects to NTO populations are expected because of cultivation of DP-915635-4 maize based on the expected levels of exposure to the IPD079Ea protein and the results of laboratory toxicity studies. ^50^; ^51–54^; ^55^; ^56,57^
